# Supplementary material for: Are carbon emissions trading and green financial instruments synergistic? -Comprehensive quantitative research based on content analysis
Source: PLoS One. 2024 Mar 7;19(3):e0298601. doi: 10.1371/journal.pone.0298601 (PMC10919601; doi:10.1371/journal.pone.0298601)
Supplement: S1 Data — (ZIP) [file pone.0298601.s001.zip › Data/地级市Z_F工作报告.zip!/║╙─╧/σo╤⌠╩╨/└┤╫╘╣1⁄2╓┌║┼í╛┬φ┐╦╩2╛▌═°í┐_2011.docx]

各位代表：

现在，我代表市人民政府，向大会作政府工作报告，请予审议，并请政协各位委员提出意见。

一、2010年及“十一五”工作回顾

2010年，在省委、省政府和市委的正确领导下，全市上下深入贯彻落实科学发展观，坚持“四个重在”实践要领，突出实施工业强市战略，真抓实干，开拓进取，濮阳经济社会发展保持了持续向好的基本态势，完成了市六届人大二次会议确定的目标任务。

———经济形势持续向好。全市生产总值774.8亿元，增长11.4%。地方财政一般预算收入30.2亿元、支出90.4亿元，分别增长19.1%和10.8%。规模以上工业增加值426亿元，增长

15.2%。

———发展后劲持续增强。全社会固定资产投资532.2亿元，增长22.5%，其中，城镇固定资产投资432.4亿元，增长21.2%；社会消费品零售总额230.7亿元，增长18.4%。

———人民生活持续改善。城镇居民人均可支配收入15138元，农民人均纯收入5077元，分别增长10.2%、15.1%。

———社会大局和谐稳定。科技、教育、文化、人口和计划生育等社会事业全面发展。公共卫生体系建设得到加强。产品质量和食品药品安全保障水平继续提高。安全生产、信访、社会治安、民族宗教等工作取得新成绩，社会大局保持稳定。

一年来，我们主要抓了以下重点工作：（一）努力在扩大需求中保持增长态势。重点项目建设取得明显成效。实施新增中央投资项目131个，完成投资5.6亿元；实施“转型升级双千工程”项目22个，完成投资27.5亿元；实施省定重点项目14个，完成投资18.3亿元；实施市定重点项目170个，完成投资120.1亿元。开工建设了投资14.7亿元的中原乙烯60万吨甲醇制20万吨烯烃、投资10.8亿元的永金化工20万吨乙二醇、投资5.6亿元的永龙化工100万吨真空盐等项目。

投资8.6亿元的中原油田文96地下储气库项目正在加快推进，投资80.2亿元、库容101亿立方米、日调峰能力3800万立方米的文23国家战略储气库项目落户我市。投资对经济增长的贡献率70.1%。消费需求持续扩大。落实国家家电下乡、汽车摩托车下乡、汽车以旧换新等政策，带动消费6.2亿元。深入实施“万村千乡市场工程”，建成农家店592家。商品房销售面积148.8万平方米，增长36.2%。消费对经济增长的拉动作用不断增强。外经外贸运行稳定。全市进出口总额4.9亿美元，增长22.9%。其中出口4.2亿美元，增长28.3%。外经指标继续保持全省领先水平。

在推进工作中，切实加强经济运行调控调节，深化企业服务，保障了企业生产正常运行。

（二）努力在优化结构中推进经济转型。规划引领作用不断增强。编制完成了资源型城市转型规划和化工、食品、林纸林板、特种玻璃及电光源等现代产业体系发展专项规划。产业集聚区建设扎实推进。开展了“产业集聚区建设加速年”活动。８个省定产业集聚区完成投资140.4亿元，其中基础设施投资28.4亿元。建成标准化厂房38.5万平方米。完成融资9.7亿元。新入驻项目121个，其中超亿元项目49个。投资20亿元的全友家私、投资7亿元的东金家具、投资3亿元的雨润200万头生猪屠宰分割、投资13亿元的永乐生物L—乳酸、投资6亿元的金苑面业、投资12亿元的韩商服装工业园等一批项目落户产业集聚区。这些项目的建设，进一步夯实了化工、机械制造等产业基础，初步形成了家具制造、食品加工、羽绒服装等产业雏形。清丰县、南乐县产业集聚区和濮阳经济技术产业集聚区入选全省50个先进产业集聚区。自主创新能力不断增强。争取国家、省科技项目57项，争取国家科技型中小企业创新基金7项。建成省级工程技术研究中心5家、市级8家。培育高新技术企业5家。专利申请量、授权量分别为958件和615件。濮耐创新能力建设专项列入国家创新能力建设项目计划。全年实现高新技术产业总产值55亿元。现代服务业发展取得新成绩。引进了投资6.6亿元的港聚源物流园区项目和投资33.3亿元的名嘉购物广场项目。

组建了市政府金融办，完善了金融服务体系，完成新增贷款和资本市场融资93亿元，占省定目标的119.4%。成功举办了“2010中国濮阳国际精品杂技魔术节”。精品杂技剧目《水秀》完成二度创作并进行商演。华晨杂技集团参加了广州亚运会的开闭幕式演出。全年接待游客313.9万人次，旅游收入4.4亿元。资源节约型和环境友好型社会建设取得新成效。完成了节能减排目标。实现了耕地占补平衡。城市空气质量优良天数90%。饮用水源地取水达标率100%。林业生态市建设扎实推进。

（三）努力在加快城镇化中促进城乡协调。启动了中心城市综合改造提升工程，推行了城市精细化管理，城市面貌明显改观，城市形象明显提升。中原路东延工程建成通车，石化路西延、新东路等道路建设进展顺利。供水管网改扩建、供气管网改扩建、集中供热改扩建、生活垃圾无害化处理场、背街小巷改造等工程完工。综合交通体系建设扎实推进。大广高速公路南乐至豫冀省界段、工业大道、大广高速公路至南乐县城快速通道建成通车。晋豫鲁铁路通道濮阳段开工建设，该路建成后将结束我市中心城区不通国铁的历史。濮范高速公路、濮清快速通道、范县新区至山东省界快速通道正在加快建设。德商高速公路范县段各项准备工作全部完成，近日将开工建设。县城和小城镇建设取得新成绩。

（四）努力在夯实基础中解决“三农”问题。发放涉农补贴9.6亿元。粮食总产251万吨，增长0.82%。新增塑料大棚、日光温室4241座。林下经济达到15.3万亩。畜牧业总产值70亿元。127家市级以上农业产业化龙头企业实现销售收入120亿元，利税8.7亿元。投资1.1亿元的金堤河干流二期治理工程获准立项，中央下达投资9070万元。完成了渠村、南小堤、彭楼三大灌区配套工程。完成了台前梁庙沟干流及马颊河北里商闸至高庄闸段治理任务。完成了第三濮清南北延工程。荣获全省“红旗渠精神杯”市级杯十一连冠。新改建农村公路253.5公里。农村劳动力转移70.2万人次，实现劳务收入63亿元。新农村建设有序推进。

（五）努力在扩大开放中增强发展活力。组织参加了国家、省举办的大型招商活动。自主开展了上海、天津、深圳等驻地招商活动。举办了中华张姓受封纪念大会、中国大型企业台资企业濮阳投资洽谈会等自主招商活动。与中石化、中石油、中国航天工业集团、中国兵器集团、华润集团等18家央企达成了战略合作意向。与中原油田、省移动公司、省联通公司、省电力公司签订了战略合作协议。全市新签市外资金项目180个，其中亿元以上项目141个。合同资金总额426亿元，到位104.5亿元；新批外商投资企业6家；实际利用外资9001万美元，增长19.9%。

（六）努力在深化改革中破解发展难题。市县两级政府机构改革全面完成。完成8户国有企业改革任务。市直机关所属企业脱钩改制启动。白条河农工商总公司整体移交河南投资集团。濮阳市商业银行挂牌营业。医药卫生体制、文化体制等改革积极推进。非公有制经济发展迅速。全市规模以上非公有制企业826家。非公有制经济完成增加值451亿元，占全市经济总量的59.4%。

（七）努力在改善民生中构建和谐濮阳。十项民生工程年度目标任务全部完成。城镇新增就业人员9.7万人，下岗失业人员再就业4.2万人。城镇登记失业率2.59%。发放小额贷款7.8亿元，扶持创业1.6万人。城乡低保应保尽保。社会保障覆盖面不断扩大，失业保险实现市级统筹。新农合参合率98.3%。保障性安居工程完成年度任务。筹资1.9亿元实施了校安工程，改造学校项目93个。新建改扩建城镇中小学校10所。普通高招本科上线率45.7%，连续5年居全省第一。实施了职业教育攻坚计划。在全省率先实现行政建制县 （区）计划生育优质服务单位全覆盖。开展了婚前免费体检。参加了省第十一届运动会，取得了总分第九名的成绩。第六次人口普查进展顺利。连续7年被省政府评为 “全省安全生产工作优秀市”。

平安建设成效明显，被省委、省政府表彰为 “平安建设先进市”。外事侨务、机关事务管理、发展研究、工商、质监、残疾人工作、市场发展、史志、地震、人防、气象、保险、邮政、通信等各项事业都取得了新成绩。大力开展国防教育，扎实做好双拥工作。驻濮解放军和武警、消防部队在支持地方发展、维护稳定等方面做出了积极贡献。

（八）努力在加强自身建设中全面履行职责。坚持 “三具两基一抓手”，扎实推进 “两转两提”，深入开展 “一创双优”集中教育活动，各级政府行政效能和服务水平进一步提升。坚持依法行政，按照法定权限和程序行使权力、履行职责，政府法制工作不断加强。自觉接受人大及其常委会监督，支持政协参政议政，办理人大代表建议139件、政协委员提案280件。

认真听取各民主党派、工商联、无党派人士的意见，支持工会、共青团、妇联等人民团体开展工作。大力推行政务公开，加强电子政务建设。加强政府廉政建设，深化财政审计监督，反腐倡廉取得新成效。加强精神文明建设，公民道德水平和城乡文明程度不断提升。

各位代表！2010年各项任务的完成，标志着我市“十一五”规划确定的目标顺利实现。“十一五”时期，我市综合经济实力不断提升。“十一五”期间，全市生产总值累计实现3022亿元，是“十五”时期的2.1倍，年均增长12%。地方财政一般预算收入累计完成122.5亿元，是“十五”时期的2.2倍，年均增长14.5%。规模以上工业增加值累计完成1601亿元，是“十五”时期的2.9倍，年均增长17.5%。全社会固定资产投资累计完成1738亿元，是“十五”时期的3.1倍，年均增长27.7%。2010年全市粮食总产251万吨，较“十五”末增加48.6万吨，连续7年保持增长。一、二、三产业结构由“十五”末的16.3∶62.7∶21.0调整为13.9∶66.5∶19.6。我市基础设施不断完善。综合交通体系建设加快推进，交通瓶颈制约得到缓解。城市公共设施逐步完善，累计完成城市基础设施投资220亿元，城市建成区面积由“十五”末的35平方公里增加到51平方公里，中心城区集中供热率达到75%，燃气普及率达到90%。县城和小城镇建设不断加快。农业基础设施建设进一步加强，渠村引黄闸、金堤河张庄提排站等一大批项目建成投用。解决了66.2万人的饮水安全问题。全市8个省定产业集聚区累计完成投资183.4亿元，产业承载能力明显增强。我市社会事业全面进步。城乡教育水平明显提升。科技进步对经济增长的贡献率49%。文化事业蓬勃发展，我市被确定为省级文化改革发展试验区。医药卫生体制改革有序推进。全市人口自然增长率控制在6.5‰以内。社会保障体系逐步健全。为民所办实事工程，件件得到落实，人民群众共享了发展成果。安全生产长效机制不断健全，信访形势保持平稳态势，社会治安防控体系得到加强，社会大局持续和谐稳定。我市可持续发展取得新成效。全面加强资源和环境保护，建立了落后产能淘汰机制，实施了造纸、化工等高污染、高耗能行业综合整治工程和生态保护重点流域综合整治工程，生态环境得到明显改善。完成了节能减排目标任务。经过五年的努力，濮阳发展站在了新的战略起点上。

上述成绩的取得，是省委、省政府和市委正确领导的结果，是人大、政协和社会各界大力支持的结果，是全市人民群策群力、团结奋斗的结果。在此，我代表市人民政府，向工作在全市各条战线的工人、农民、知识分子、干部、人民解放军指战员、武警消防官兵、公安民警以及所有关心、支持濮阳发展的各界人士致以崇高的敬意，表示衷心的感谢！各位代表！在肯定成绩的同时，我们也清醒地认识到，我市经济社会发展中还存在不少困难和问题：一是 “十一五”时期虽说与自己比有发展，但放在全省大局中，几个主要经济指标的总量、人均及增幅在全省处于平均水平以下，赶超任务艰巨，发展压力很大。二是三次产业发展不协调，工业结构单一，基础薄弱，支撑作用不强，服务业发展滞后。三是重大项目谋划建设不足，特别是在建项目规模小、数量少，缺乏战略支撑型项目。四是中心城市基础设施欠账多，功能不完善，辐射带动能力不强。五是县城和小城镇承载能力不强，县域经济基础薄弱。六是一些政府工作人员思想观念不够解放，工作作风不够扎实，服务意识不强，办事效率低下。对此，我们一定高度重视，认真解决。

二、 “十二五”发展总体思路和目标任务

《濮阳市国民经济和社会发展第十二个五年规划纲要》是指导濮阳今后五年发展的行动纲领。今后五年，我市经济社会发展的总体思路是：以科学发展为主题，以加快转变经济发展方式为主线，以 “四个重在”为实践要领，牢牢把握中原经济区重要出海通道、中原经济区与环渤海经济圈衔接融合的前沿、省际交汇区域性中心城市的战略定位，紧紧围绕建设富裕和谐美丽新濮阳中心任务，突出加快发展，坚持统筹协调，深化改革开放，持续开展 “一创双优”活动，强力实施工业强市战略，加快推进城镇化和农业现代化，着力改善民生，着力维护社会和谐稳定，力争经济发展速度两年时间赶上全省平均水 平，三年时间超过全省平均水平，五年时间进入全省先进行列。

“十二五”时期经济社会发展的主要目标是：———综合实力明显增强。到2015年，地区生产总值、财政一般预算收入翻一番以上；全社会固定资产投资年均增长22%以上，投资结构进一步优化；消费水平逐步提升；利用外资明显增加；区域竞争力明显增强。

———经济转型步伐加快。以传统优势产业和战略性新兴产业为主导的产业结构不断优化。到2015年，高新技术产业增加值占工业增加值的比重达到20%；科技进步对经济增长的贡献率达到54%；服务业规模不断壮大；农业现代化水平明显提高；非公有制经济占全市生产总值的64%；城镇化率达到

48%。

——人民生活水平显著提高。城镇居民人均可支配收入、农民人均纯收入年均增长9%和11%；年新增就业岗位7万个，城镇登记失业率控制在4.5%以内；贫困人口数量明显减少。

——社会建设全面加强。教育普及程度、层次明显提高；基本建立覆盖城乡的社会保障体系、社会救助体系和社会预警应急体系；人口自然增长率控制在6.5‰以内；社会事业全面进步，公共服务体系更加健全，民主法制、精神文明建设全面加强。

——生态环境明显改善。节能减排完成省定目标；城市污水集中处理率达到86%以上；垃圾无害化处理率达到95%以上；林木覆盖率达到28%以上；城市空气质量不断提高；初步建立生态良好、资源支撑、环境安全、自然和谐的生态市框架，可持续发展能力不断增强。

实现上述目标任务，必须强化四种理念：强化加快发展的理念。始终把发展作为第一要务，当成第一要义，采取超常规措施，加压驱动，提速晋位，加快发展。在加快发展中促转型，在转型中加快发展。强化统筹协调的理念。坚持以工业化为支撑、城镇化为引领、农业现代化为基础，实现 “三化”协调发展。坚持统筹经济社会协调发展，推进社会事业全面进步。坚持统筹人与自然和谐发展，更加注重资源节约型和环境友好型社会建设，走可持续发展之路。强化改革开放的理念。

坚持用改革的办法和市场经济的方法破解发展中遇到的难题，形成有利于濮阳发展的体制机制与政策环境。坚持以开放促发展，把扩大对外开放作为借助外力、带动全局的战略举措，千方百计引进资金、技术和人才，增强加快濮阳发展的动力。强化改善民生的理念。坚持执政为民、以人为本，把实现和维护群众的根本利益作为出发点，把为民办实事、做好事、解难事作为着力点，切实改善民生。

实现上述目标任务，必须抓好六项战略重点：着力实施工业强市战略。以产业集聚区建设为载体，以现有企业改造提升和承接产业转移为途径，实施一批重大工业项目，盘活存量，扩大增量，着力构建新型工业化体系，推进工业经济快速发展，充分发挥工业化对濮阳发展的支撑作用。着力推进城镇化进程。坚持走新型城镇化道路，充分发挥中心城市核心带动、县城骨干支撑、小城镇支点吸纳作用，以产兴城、以城带乡，产城融合、相互促进，不断增强城镇承载和服务能力。着力调结构促转型。围绕资源型城市经济转型这一战略任务，做大做强优势战略支撑产业，积极培育战略性新兴产业，加快油气产业主导向综合性产业多元支撑转变，促进产业结构转型升级。

着力打造区位新优势。加强综合交通体系建设，发挥濮阳地处三省交汇处的地理优势，发挥中原经济区与环渤海经济圈衔接融合的前沿效应，发挥濮阳距离出海港口较近的独特优势，实现与发达地区在交通和产业等方面的有效对接。着力深化改革开放。坚持市场化改革方向，努力破解土地、资金、人才等瓶颈制约和体制机制障碍。同时，着眼国际国内两个市场，强力实施开放带动战略，持续开展大招商活动，全面提升对外开放水平。着力改善民生。坚持发展为了人民，发展成果人民群众共享，更加注重增加城乡中低收入居民的收入，扩大社会保障覆盖面，提升公共服务水平，促进人的全面发展。

各位代表！“十二五”规划的宏伟蓝图已经绘就。我们坚信，在省委、省政府和市委的坚强领导下，只要全市人民同心同德，顽强拼搏，勤劳勇敢的濮阳人民就一定能够战胜前进道路上的一切艰难险阻，我市 “十二五”规划的宏伟蓝图一定能够实现，濮阳的明天一定会更加美好！

三、2011年政府工作的奋斗目标和主要任务

2011年是实施 “十二五”规划的开局之年。开好头、起好步，事关今后五年发展大局。当前，我们面临着加快发展的诸多有利条件和重要机遇。世界经济缓慢复苏，我国经济发展长期向好的趋势没有改变。今年，国家实施积极的财政政策和稳健的货币政策，为我们调整优化经济结构、转变发展方式提供了难得的环境。国内外发达地区产业转移的加快，为我们承接产业转移提供了新的历史机遇。中原经济区正式纳入 《全国主体功能区规划》，上升到国家战略层面，有利于我们更好地发挥区位优势，在更大范围内集聚要素，在更广领域内拓展市场。随着 “一创双优”活动的持续开展，全市上下思想观念不断创新，干部作风不断转变，发展环境不断优化，形成了好的发展气势，为加快发展奠定了思想和作风基础。同时也要看到，我市经济发展仍然面临着许多困难和挑战。世界经济虽然保持复苏状态，但宏观经济回升的基础依然薄弱；国家宏观调控政策进入转换期，土地、资金等要素供给趋紧。为此，我们既要增强机遇意识、赶超意识，又要增强忧患意识、责任意识，始终扭住发展不放松，变压力为动力，化挑战为机遇，强力推进濮阳加快发展。

今年经济社会发展主要预期目标是：生产总值增长10%，地方财政一般预算收入增长12%，全社会固定资产投资增长22%，社会消费品零售总额增长17%，外贸出口增长25%，实际利用外商直接投资增长25%，城镇居民人均可支配收入增长9%左右，农民人均纯收入增长10%左右，居民消费价格涨幅控制在4%左右；人口自然增长率控制在6.5‰以内；城镇登记失业率控制在4.5%以内；节能减排完成省定目标。

为实现上述目标，今年重点抓好以下十个方面的工作。

（一）着力抓好需求拉动，保持经济持续较快发展。紧紧抓住扩大消费、投资拉动、增加出口等关键环节，加强经济运行调控调节，为经济持续较快发展创造条件。扩大消费需求。

落实国家、省调整收入分配关系的政策措施，增强居民消费能力。引导住房、汽车合理消费。落实好家电、摩托车下乡和家电以旧换新政策。继续开展 “万村千乡市场工程”建设。推进放心早餐工程、家政服务工程，加快发展社区商业、物业、家政等服务性消费。发展文化旅游消费、老龄消费、休闲健身消费等多样化消费。整顿和规范市场秩序，保持市场物价基本稳定，维护消费者权益。扩大投资规模。重点在产业升级、交通 能源、城乡建设、社会事业等领域，认真谋划一批、建设一批、储备一批支撑、辐射、带动作用强的基础设施和基础产业项目。集中力量抓好246个重点项目，概算投资733.6亿元，年度计划投资335.8亿元。其中亿元以上项目147个，5亿元以上项目35个，10亿元以上项目17个。年内全市在建房屋面积力争突破1000万平方米。完善重点项目推进机制，实行市级领导分包重点项目以及周调度、月汇报、季观摩、年评比的项目推进制度，着力打造重点项目建设 “绿色通道”。扩大对外贸易。把承接产业转移作为带动出口的主要途径，大力引进相关出口型企业，加快培育出口基地，力争高新区石油化工产品出口基地和华龙区石油钻采设备出口基地列入省级出口基地，加快培育提升濮阳县电光源出口基地和台前县羽绒制品及服装出口基地。加快商检办事处建设，力争年内建成投用；协调郑州海关，争取在我市设立海关分支机构，做好与日照港、黄骅港等出海口的对接，谋划推进 “无水港”建设。加强经济运行调控调节。坚持经济运行月例会、季分析制度，密切关注国家政策调控动向，跟踪分析我市经济形势变化。加强重点行业、重点企业监测，做好煤电油气运供需协调，保障要素供应。加大银企合作力度，每季度组织一次银企对接洽谈会。采取有力措施，进一步加强财税工作。

深化企业服务。坚持抓服务就是促发展、增效益的理念，建立健全政策导向、市场开拓、融资服务、困难企业帮扶等长效机制，推动企业服务常态化、制度化、精细化，营造企业发展良好环境。

（二）突出实施工业强市战略，增强工业经济对濮阳发展的支撑作用。坚持 “项目带动、工业支撑、特色主导、集聚发展”的思路，以开展工业强市杯竞赛活动为载体，大力实施工业发展 “六大行动计划”，推进工业经济快速发展。实施重点工业项目推进行动计划。初步谋划投资超千万元的重点工业项目160个，概算总投资290亿元，年度计划投资160亿元。其中投资5000万元以上的项目123个，投资亿元以上的项目75个。建成中原乙烯60万吨甲醇制20万吨烯烃、永金化工20万吨乙二醇、永龙化工100万吨真空盐和永乐生物L—乳酸一期12万吨淀粉等项目；开工建设恒润石化20万吨碳四芳构化、硕维电子30万台电脑和200万套LED灯具、林氏化学40条医用手套生产线、信宇工业园1500台节能型抽油机、新南方家私20万套家具制造等项目；积极推进龙丰热电2×60万千瓦热电机组、龙丰纸业二期工程等项目前期工作。实施产业集聚区建设行动计划。开展 “产业集聚区建设提升年”活动。完善集聚区内道路、电力、供排水等基础设施，推进标准化厂房、办公商务、仓储用房建设，为项目入驻提供条件。立足集聚区产业定位和发展优势，策划和组织集聚区专题招商，积极承接产业转移，引进行业龙头企业，年内完成各产业集聚区主导产业骨架的搭建，夯实产业基础。大力发展工业房地产、人才培训、现代物流等生产性服务业，支持建设科技研发、检测检验、信息咨询等公共服务平台，提高产业集聚区承载能力和吸引力。统筹产业集聚区与城镇化发展的对接，逐步形成以产兴城、以城促产、产城融合发展的格局。坚持 “月分析、季观摩、年评比”工作制度，实现产业集聚区建设的全面提升。支持高新区、工业园区加快发展步伐，使其尽早建设成为濮阳对外开放的窗口和经济发展的动力基地。年内全市8个产业集聚区完成投资160亿元，其中基础设施30亿元、标准化厂房10亿元、工业项目120亿元，实现营业收入500亿元。

实施大企业培育行动计划。大力支持中原油田、中原乙烯及河南煤化集团、河南投资集团在濮企业做大做强，继续抓好濮耐、蔚林化工、宏业化工等地方骨干企业发展，推动我市企业与中央、省属企业和行业大型企业的合作，逐步培育一批主业突出、核心竞争力强的大型企业集团。实施中小企业提升行动计划。支持中粮面业、贝英数控、中博化工等中小企业优化产品结构、提升产业层次，强化管理创新，增强中小企业综合竞争力。积极推进油田改制企业 “退城入园”工作，促其调整结构、膨胀规模。争取年内新增30家规模以上工业企业。实施企业上市助推行动计划。筛选一批上市后备企业，在政策和资金方面给予重点扶持，帮助其尽早上市。力争年内实现蔚林化工上市，做好泓天威药业、中拓管道、宏业化工、绿能高科等企业的上市准备。实施百名优秀企业家培养行动计划。自今年起，每年择优选拔一批企业高级经营管理人才，采取多种形式培训，努力打造一支具有世界眼光、战略思维、创新精神和经营能力的优秀企业家队伍。

（三）坚持统筹城乡协调发展，增强城镇化对濮阳发展的引领作用。提升城乡建设水平，增强城市的辐射力和承载力，加快推进城镇化进程。综合提升建成区。以实施中心城市综合改造提升工程为抓手，通过建设、改造、管理、教育四条途径，实施城市精细化管理，不断完善城市功能，提升城市形象。继续实施 “打通断头路工程”，年内打通石化中路、古城路等断头路。全面改造颐和路、桃园路等支背小巷。全面实施建成区人行道破损路面维修改造工程。建设黄河路、任丘路、建设路等污雨水泵站，汛期前完成城区积水点改造。改建环保压缩式中转站5座、公厕10座。建设榆济线天然气引接工程。

加快推进长庆片区综合开发。加快城中村改造步伐，争取三年内全部纳入城市规划和管理体系。对于建成区烂尾工程、在建工程，明确责任主体，限时完成。建立迎检工作台账，确保国家卫生城市顺利通过复检。加快开发新城区。抢抓省支持省辖市建设新区的战略机遇，积极推进濮阳新区规划编制工作。按照 “一年打基础，两年见形象，三年成规模”的工作目标，今年集中力量抓好濮阳新区起步区——濮北新区建设。重点推进路网建设和濮北新区水库建设。开工建设开州路北延、濮上路北延、昆吾路北延、卫河路北延、历山路北延和五一路西延、绿城路改造、开德路、北环城路，年内完成节点目标，初步形成濮北新区 “五纵四横”路网框架。以开工建设濮北新区水库为契机，规划建设城市水系，着力打造独具濮阳特色的水系景观。加强县城和小城镇建设。加强县城基础设施建设，加快县城城中村改造，完善服务功能，拓展发展空间，提升县城承载能力。加快濮阳县城、清丰县城与中心城市的对接，逐步推进三城一体化。加快濮阳县老城区改造，实现与中心城市在道路等市政设施方面的先期融合。推进清丰县新城区向南发展，实现基础设施和公共服务设施与濮北新区的对接。统筹推进南乐县、范县、台前县作为独立城市组团加快发展。着力解决进城务工人员的就业、安居、子女就学、社会保障、户籍管理、承包土地、宅基地和集体财产权益等问题，推进农村人口向县城集聚。在基础设施建设、产业发展、项目布局、金融服务等方面制定扶持政策，支持县域经济加快发展。支持各县 （区）依托自身资源禀赋，以产业集聚区为载体，大力招商引资，培育1至2个特色产业集群，打造一批竞争力强的县域骨干企业，实现县域经济发展新跨越。加大撤乡建镇工作力度，支持乡镇发展特色优势产业，逐步做大城镇规模，提高辐射带动能力。

加快构建综合交通体系。年内建成濮范高速公路、市城区至清 丰县城和范县新区至山东省界快速通道。推进晋豫鲁铁路通道濮阳段建设。开工建设德商高速公路范县段、G106濮范高速公路至东明黄河桥段升级改造、S212清渠线濮阳县南关至庆祖段改造工程。配合省做好郑州经濮阳至济南客专前期工作。

推进南林高速公路东延工程、濮阳至东明高速公路、范县—台前—梁山高速公路和德商高速公路至台前县城快速通道前期工作。

（四）大力发展现代农业，夯实 “三化”协调发展基础。

坚持把解决好 “三农”问题作为全部工作的重中之重，大力发展现代农业，充分发挥农业现代化在 “三化”协调发展中的基础作用。落实强农惠农政策。财政支出重点向农业农村倾斜。

按标准落实好农业各项补贴。稳步提高重要粮食品种最低收购价。加大政策性金融、保险对 “三农”支持力度。保障农产品供给。积极推进优质、高效、有机、无公害粮食生产基地项目建设，按照提高单产、保障总产的要求，粮食综合生产能力稳定在240万吨以上。加快高标准农田建设，重点实施高产稳产田巩固提升、中低产田改造、土地整治三大工程。落实 “菜篮子”市长负责制，有效保障 “菜篮子”产品生产供应、质量安全和价格平稳。加大农业结构调整力度。建设一批特色产业基地。发展设施农业，在完成 “4293”工程 （新增设施农业面积4.2万亩，建设现代设施农业示范园区93个）的基础上，再新建、扩建设施农业示范园区80个，设施农业总面积发展到30万亩。发展现代畜牧业，新建和改建标准化养殖场区40个。

新发展林下经济15万亩，实现产值5亿元。提升农业产业化水平。重点培育壮大127家市级以上农业产业化龙头企业，年销售收入超亿元的达到22家，超5亿元的达到2家。建成福润10万头肉牛、雨润200万头生猪屠宰加工项目，推进大用1亿只肉鸡产业化项目建设。发展农民专业合作社和行业协会。推行 “超市+专业合作社+基地”模式，打造农副产品进城、进超市的快捷通道。推广先进适用农机具。完善农技推广、动植物疫病防控、气象灾害防御等服务体系。强化农业基础设施建设。实施黄河标准化堤防建设、金堤河干流二期治理、中小河流治理、灌区配套工程。推进国家新增千亿斤粮食工程、南水北调濮阳受水区工程建设。实施 “5+3”林业工程，完成廊道绿化、城郊绿化、产业集聚区绿化、农田林网建设、林果基地建设五项重点工程，搞好村镇、学校、企业三个层面绿化，完成植树2024万株，工程造林16万亩。加快农村电网改造升级。积极稳妥推进新型农村社区建设。中心城市近郊、产业集聚区和城市新区的村庄，按照规划先行、就业为本、群众自愿、量力而行的原则，积极推进新型农村社区建设。实施新农村建设示范村带动工程，统筹推进新农村建设，塑造环境整洁、秩序井然、生态良好、乡风文明的农村新形象。

（五）加快发展第三产业，努力打造新的经济增长点。加快发展现代服务业，不断扩大第三产业规模，全力提升第三产业质量和水平。大力发展文化旅游产业。推进文化改革发展试验区建设。今年重点打造推出以中国濮阳国际杂技文化产业园、东北庄原生态杂技文化园、蓝宝石温泉生态休闲度假园为主体的 “濮阳杂技休闲旅游”精品线路，统筹谋划清丰单拐、台前将军渡、范县颜村铺 “红色旅游”和以濮阳县渠村分洪闸、范县毛楼生态旅游区、台前张庄黄河大闸、黄河大堤绿化带和黄河湿地为重点的 “黄河休闲旅游观光带”。大力发展商贸物流业。加快中心城区现代服务业园区建设，丹尼斯濮阳商城年内建成开业，开工建设万利财富广场二期工程、科技信息服务大楼、企业总部大厦、银座国际酒店、华阳国际公馆、正大家居二期工程等项目。加快推进名嘉购物广场项目。大力发展食品冷链、医药、钢材、家电等行业物流。推进豫北商贸物流园区建设。加快中原油田文96地下储气库建设，开展文23国家战略储气库前期工作。规划建设综合能源物流中心。加强信息网络服务。积极推进 “三网”融合，加快3Ｇ网络建设。

开工建设濮阳移动通信分公司生产楼、综合办公楼和濮阳电信通信枢纽楼。基本完成有线电视数字化整体转换和双向改造。

加快打造金融生态区。出台促进金融业发展的政策措施，引进股份制银行落户濮阳。支持市商业银行拓展服务领域，年内建成濮阳县分支机构，两年内实现五县全覆盖。支持各县 （区）引进符合条件的银行机构组建村镇银行，加快推进农村信用社改制为商业银行。整合信用担保机构，打造3至5家较大规模的投资担保公司。完善金融和信用担保体系，加强监管，严厉打击违法犯罪行为，构建生态、安全、高效、繁荣的金融集聚高地。

（六）持续抓好大招商工作，增强加快发展的活力。深入实施开放带动战略，坚持一手抓招商引资，一手抓项目落地，借力发展，借势发展。着力抓招商引资。组织参加好国家、省举办的经贸活动。重点举办好自主招商活动。搞好专题招商、驻地招商、小分队招商，突出抓好园区招商、以商招商。强化招商选资引智理念，注重引进科技含量高、经济效益好、带动能力强、集聚效应好的项目，注重引进技术、人才和先进的管理经验。紧盯国内外500强和行业龙头企业，积极引进战略投资者。深化与央企的战略合作，重点抓好已签约协议或合作项目的落实。组织实施《濮阳市人民政府中国石化中原油田战略合作与发展框架协议》，在产业发展、基础设施、社会事业和民生工程等方面，双方共同谋划建设一批合作项目，实现油地融合、共赢发展。借助中原油田加强与中石化集团的战略合作，规划建设中石化产业园。巩固和发展中石油对范县、台前两县产业化帮扶工作，推进中石油产业扶贫开发示范区建设。年内完成引资合同额500亿元，实际利用市外资金150亿元。着力抓项目落地。全市新开工亿元以上项目80个，其中5至10亿元项目20个，10亿元以上项目10个。健全招商引资考核奖惩机制，提高合同签约率、项目落地率、资金到位率。

（七）积极推进改革创新，激发加快发展的动力。坚持用改革创新的办法解决前进中的困难和问题，努力在重点领域和关键环节取得新突破。不断深化改革。引进战略投资者，推进国企改革，健全国有资本有进有退、合理流动机制。推进市直机关所属企业脱钩改制。按照上级统一部署，推进事业单位人事、工资、职称制度改革和公益性文化事业单位改革。继续推进农村综合改革。健全农村土地承包经营权流转机制，加快农村土地流转。不断创新水利发展体制，推进水利投融资体制改革。

协调推进集体林权制度、县级供销社体制改革。积极稳妥推进公安警务机制改革。深化医药卫生体制改革。加快推进市天然气公司、热力公司等公益性事业单位改革。认真落实国家和省在财税、金融、投资、价格等方面的改革措施。创新体制机制。采取措施，着力破解用地指标供需矛盾突出、土地储备滞后、土地利用粗放和企业贷款难、项目融资难、基础设施投入不足等瓶颈制约，理顺教育管理、城市管理等方面的体制。大力发展非公有制经济。落实完善促进非公有制经济发展的政策措施，支持民间资本进入基础设施、资源开发、公用事业等领域，推进非公有制经济持续、快速、健康发展。

（八）加强节能减排和自主创新，增强可持续发展能力。落实节能减排目标问责制和“一票否决”制。健全激励和约束机制，促使企业加快技术改造和产品结构调整。继续实施八大节能工程，推进高耗能设备更新改造。严格项目准入审查，从源头上控制能耗不合理增长。推行重点建设项目污染物总量调配直通车制度，淘汰落后产能。全面推广清洁生产，切实抓好循环经济试点。开展环保生态示范创建活动。加强耕地和基本农田保护。加强农村环境保护，控制农村面源污染。启动油区土地污染治理。加快城镇污水处理设施管网配套。加快建设市第二污水处理厂和市污水处理厂脱氮改造二期工程。全面实施水环境生态补偿，着力建设资源节约型、环境友好型社会。

大力推进自主创新。加快推进市科技企业孵化器建设，年内建成科技创业服务楼。争取省级以上科技项目25项，新增高新技术企业3家，建立院士工作站1家，新建省级工程技术研究中心3家，市级工程技术研究中心5家。实施 “质量兴市”战略，做好市长质量奖评选工作。加快市产品质量综合检测实验楼建设，积极推进国家电光源质量监督检验中心项目建设。实施知识产权优势培育工程，打造著名商标、特色品牌，促进企业技术进步，提高产品质量和竞争力。

（九）切实改善民生，促进濮阳和谐发展。始终把改善民生作为一切工作的出发点和落脚点，大力发展各项社会事业，让改革发展成果惠及全市人民。集中力量实施好民生工程。（1）改善城乡居民生产生活条件。抓好偏僻、贫困乡镇农村公路建设，新改建农村公路253公里，完善县县畅、乡乡联、村村通公路网络。改造危桥14座。解决20万以上农民的安全饮水问题。实施马颊河、濮水河、第三濮清南干渠综合整治，让水活起来、清起来。（2）搞好扶贫开发，完成50个村的整村扶贫推进任务。扶持黄河滩区开发建设。（3）加强保障性住房建设。开工建设经济适用住房30万平方米，竣工20万平方米，廉租住房保障户数达到10000户以上，完成工矿棚户区改造5200户、40万平方米。（4）减轻农民负担。今年起，免除农业水费。（5）提高社会保障水平。继续扩大社会保障覆盖面，建立和完善社会保障体系。全面推进企业工 资集体协商工作。继续提高企业退休人员基本养老金。将具有城镇户籍的未参保集体企业退休人员全部纳入基本养老保险。做好养老保险关系转移接续和医疗保险异地就医工作。将未就业城镇居民育龄妇女住院分娩医疗费用纳入居民医保基金支付范围。提高社会保险统筹层次，实现医疗保险、工伤保险市级统筹。

搞好清丰县、濮阳县新农保试点。（6）进一步扩大就业。城镇新增就业7万人以上。失业人员再就业1.8万人。做好就业困难人员再就业帮扶工作，动态消除零就业家庭。新增农村劳动力转移就业2万人。发放小额担保贷款4亿元以上，支持2万名劳动者特别是高校毕业生、下岗失业人员和农民工创业。应届高校毕业生就业率达到80%以上。（7）强化公共卫生服务。做好疫病防控工作。做好公立医院改革试点，加快基层医疗卫生服务体系建设。

建成市中医院新病房楼、市妇幼保健院妇女儿童保健中心大楼。

开工建设市精神病医院病房楼、眼科医院迁建项目。谋划推进市人民医院扩建工作。（8）实施惠民殡葬政策。免除市城区基本殡葬服务费。改扩建市殡仪馆。（9）完善公共文化、科技、体育设施。整合利用市文化艺术中心，做好博物馆布展、群艺馆搬迁工作。开工建设市杂技学校迁建项目。实施文化惠民工程，新建、改扩建33个乡镇综合文化站，建成农家书屋656家。实施舞台艺术送农民、农村电影放映工程。完善市气象科技馆。完成市体育馆主体工程。（10）切实维护公共安全。推动安全生产源头治理，继续强化危险化学品、烟花爆竹、建筑施工和石油天然气开采等重点领域整顿，开展道路交通和消防安全专项整治，坚决遏制重特大事故发生；继续开展食品药品安全专项整治，严厉打击食品生产领域制售假冒伪劣，及时查处食品质量安全违法违规案件。需要说明的是，我市将在省“十项民生工程”确定后，及时对接，公布实事项目和具体内容，认真抓好落实。统筹发展各项社会事业。优先发展教育。组织实施《濮阳市中长期教育改革和发展规划（2010—2020年）》，努力办人民满意教育。大力推进素质教育。鼓励社会力量兴办教育。积极推进义务教育办学改革，均衡发展义务教育。不断提升高中阶段教育、高等教育质量。制定实施学前教育三年行动计划，有效缓解“入园难”问题。新建、改扩 建一批城区中小学。完成中小学校舍安全工程。启动农村标准化学校建设工程。规划建设职业教育园区。大力实施人才强市战略，组织实施 《濮阳市中长期人才发展规划纲要（2010—2020年）》，加快建立人力资源保障机制。统筹做好人口和计划生育工作，稳定低生育水平。繁荣发展文化事业，加强文艺精品创作，丰富群众文化生活。广泛开展全民健身活动。统筹兼顾，做好外事侨务、民族宗教、残疾人工作、发展研究、机关事务管理、统计、保险、地震、人防、史志、市场发展、工商、邮政等工作。确保社会大局和谐稳定。加强和改进群众工作，推进社会矛盾化解、社会管理创新、公正廉洁执法三项重点工作。健全信访工作责任制，畅通群众利益诉求表达渠道。完善应急管理机制，积极预防和妥善处置群体性事件和突发公共安全事件。加强流动人口管理。强化社会治安综合治理，依法防范和严厉打击违法犯罪活动。加强精神文明和民主法制建设。积极争创全国文明城市，深入开展精神文明创建活动，提高公民文明素质和城乡文明程度。完善基层民主建设。广泛开展普法教育，提高全民法律素质。充分听取各民主党派、工商联和无党派人士的意见，支持工会、共青团、妇联等人民团体参与社会管理和公共服务。加强国防教育和国防后备力量建设，支持驻濮解放军、武警消防部队、民兵预备役部队建设。加强军政军民团结，军民一心，军政和谐，携手开创双拥共建新局面。

（十）切实加强政府自身建设，提高保障发展能力。坚持好“三具两基一抓手”，扎实推进 “两转两提”，以持续开展 “一创双优”活动为动力，加快政府职能转变，做到为人民服务、对人民负责、受人民监督、让人民满意。转变职能。围绕服务型政府建设，加快行政管理体制改革，推进政府职能向更加注重强化社会管理和公共服务转变，提高公共服务质量和效率。

进一步减少和规范行政审批事项，简政放权，激发市场主体的活力。深化政务公开，切实保障人民群众的知情权、参与权、表达权和监督权。依法行政。严格依照法定权限和程序管理经济社会事务，落实行政执法责任制，规范行政执法行为。严格执行人大及其常委会的决议、决定，依法接受其法律监督和工 作监督，主动接受人民政协的民主监督，支持政协参政议政，认真办理人大代表建议和政协提案。广泛接受社会公众和新闻舆论监督，强化监察、审计等专门监督。落实廉政建设责任制，确保权力规范运行、阳光运行，建设廉洁高效政府。注重民生。坚持以人为本，关注民情，顺从民意，集中民智，维护民利，千方百计解决好事关群众切身利益的问题。完善重大事项公众参与、专家论证、政府决策相结合的决策机制，不搞形象工程，不图虚名，不做有损人民群众利益的事。倡导勤俭节约之风，杜绝浪费，严格规范出国（境）经费、车辆购置及运行费、公务接待费等支出，把有限的资金用于办好顺民意、解民忧、惠民生的实事上。优化环境。严格执行首问负责、限时办结、责任追究等制度，切实提高行政效能、工作效率和服务水平。加强效能监察，着力治懒、治庸、治乱，切实解决行政不想作为、不会作为、胡乱作为等问题。严厉打击干扰项目施工及企业正常生产经营活动的违法行为，严肃查处推诿扯皮、吃拿卡要、失职渎职、乱收费、乱罚款等案件，为濮阳发展营造更加优良的环境。务求实效。坚持“三种意识”、“六超精神”，出色履行岗位职责。在推动工作中注重运作，注重实效，不说空话，不说大话，说到做到，说好做好。高度重视调查研究，深入实际、深入基层、深入群众，问政于民、问需于民、问计于民，使发展思路、工作部署、政策措施更加符合濮阳实际，以正确的政绩造福于民。进一步完善绩效考核机制，强化督导检查，严格对重点工作的考核，切实提升执行力，以良好的政风取信于民。

各位代表！2011年是深化改革、加快发展的一年，是克难攻坚、奋勇争先的一年。加快濮阳发展，天当其时！我们坚信，任何挑战都动摇不了龙乡儿女创造美好未来的坚定信念，任何困难都阻挡不了濮阳人民豪迈矫健的前进步伐！让我们在省委、省政府和市委的坚强领导下，依靠和团结全市人民，万众一心，奋发图强，扎实工作，确保“十二五”开好局、起好步，奋力创造无愧于时代、无愧于历史、无愧于人民的一流业绩，奋力谱写建设富裕和谐美丽新濮阳的宏伟篇章！
